# Regioselective synthesis and characterization of monovanadium-substituted β-octa­molybdate [VMo_7_O_26_]^5−^

**DOI:** 10.1107/S205322961900620X

**Published:** 2019-06-07

**Authors:** Lukáš Krivosudský, Alexander Roller, Annette Rompel

**Affiliations:** a Universität Wien, Fakultät für Chemie, Institut für Biophysikalische Chemie, Althanstrasse 14, Wien 1090, Austria; b Comenius University in Bratislava, Faculty of Natural Sciences, Department of Inorganic Chemistry, Ilkovičova 6, 842 15 Bratislava, Slovakia; c Universität Wien, Fakultät für Chemie, Zentrum für Röntgenstrukturanalyse, Währinger Strasse 42, 1090 Wien, Austria

**Keywords:** polyoxometalate, POM, octa­molybdate, vanadium, ^51^V NMR spectroscopy, crystal structure

## Abstract

The monovanadium-substituted polyoxometalate anion [VMo_7_O_26_]^5−^, exhibiting a β-octa­molybdate archetype structure, was selectively prepared as penta­potassium [hexa­ikosaoxido(hepta­molybdenumvanadium)]ate hexa­hy­drate, K_5_[VMo_7_O_26_]·6H_2_O (**VMo_7_**), by oxidation of a reduced vanadomol­yb­date solution with hydrogen peroxide in a fast one-pot approach. X-ray structure analysis revealed that the V atom occupies a single position in the cluster that differs from the other positions by the presence of one doubly-bonded O atom instead of two terminal oxide ligands in all other positions. The composition and structure of **VMo_7_** was also confirmed by elemental analyses and IR spectroscopy. The selectivity of the synthesis was inspected by a ^51^V NMR investigation which showed that this species bound about 95% of V^V^ in the crystallization solution. Upon dissolution of **VMo_7_** in aqueous solution, the [VMo_7_O_26_]^5−^ anion is substanti­ally decomposed, mostly into [VMo_5_O_19_]^3−^, α-[VMo_7_O_26_]^4−^ and [V_2_Mo_4_O_19_]^4−^, depending on the pH.

## Abstract 2

The monovanadium-substituted β-octa­molybdate [VMo_7_O_26_]^5−^ was prepared by a one-pot approach using peroxido complexes of vanadium. ^51^V NMR spectroscopy confirmed the high selectivity of the synthesis.

## Introduction   

**Figure 1:**
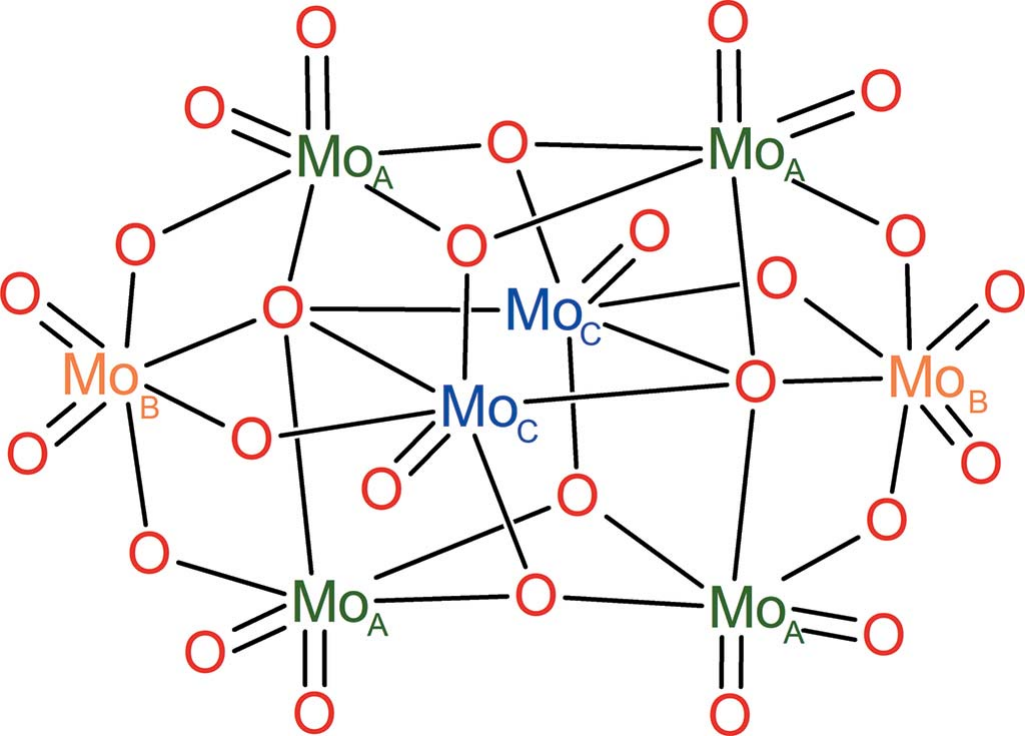
Schematic representation of the structure of the β-octa­molybdate anion [Mo_8_O_26_]^4−^. Chemically non-equivalent Mo atoms are shown in different colours: Mo_A_ green, Mo_B_ orange, Mo_C_ blue and O red.

Polyoxometalates (POMs) of W, Mo and V represent an important group of inorganic metal–oxide clusters (Pope, 1983 ▸) whose structural variability gives rise to an exceptionally wide range of applications in catalysis (Wang & Yang, 2015 ▸), magnetism (Clemente-Juan *et al.*, 2012 ▸), redox processes (Gumerova & Rompel, 2018 ▸) and materials chemistry (Song & Tsunashima, 2012 ▸), as well as in biological chemistry (Bijelic & Rompel, 2015 ▸, 2017 ▸; Molitor *et al.*, 2017 ▸; Fu *et al.*, 2015 ▸; Bijelic *et al.*, 2018 ▸, 2019 ▸). Particularly inter­esting are photoactive POMs with applications in water splitting, the photooxidation of organic pollutants, photoreductive CO_2_ activation and H_2_ generation (Streb *et al.*, 2019 ▸). Vanadium-containing POMs are a promising subgroup of photocatalysts. A V^V^ centre acts as a more efficient light absorber in comparison to Mo^VI^/W^VI^; moreover, it may easily promote a photoredox reaction *via* its photoreduction to a V^IV^ species. Substitution of some Mo or W atoms in molybdates and tungstates may therefore lead to enhanced photocatalytic properties (Streb, 2012 ▸). Substitution of one Mo atom in a Linqvist-type hexa­molybdate, [Mo_6_O_19_]^2−^, by vanadium leads to enhanced photocatalytic degradation of a model organic dye under both aerobic and anaerobic conditions caused by a low-energy O→V LMCT (ligand-to-metal charge transfer) transition in [VMo_5_O_19_]^3−^ (Tucher *et al.*, 2012 ▸). However, the controlled synthesis of mixed vanadomolybdates and vanadotungstates remains a serious challenge. Simple mixing of addenda-atom precursors leads to a complicated equilibria of several species (Pope, 1983 ▸; Howarth *et al.*, 1991 ▸). The β-octa­molybdate structure [Mo_8_O_26_]^4−^ (Fig. 1 ▸) is one of the main components in H^+^/OH^−^/MoO_4_^2−^ systems under acidic conditions, yet only the disubstituted vanadium derivative [V_2_Mo_6_O_26_]^6−^ is commonly known in the literature and has been structurally characterized (Nenner, 1985 ▸; Fei *et al.*, 2015 ▸; Li *et al.*, 2011 ▸). The two V atoms occupy chemically equivalent positions, denoted Mo_C_. Very recently, a monovanadium-substituted derivative was prepared as H_4_K_2_Na_2_(H_2_O)_4_(C_12_H_12_N_4_O_2_)[VMo_7_O_26_]·10H_2_O (Zhao *et al.*, 2018 ▸). In this case, the V atom was claimed to be statistically distributed in all positions of the parent β-octa­molybdate anion.

In the current work, we present a regioselective synthesis of a new isomer of [VMo_7_O_26_]^5−^ in which the V atom occupies only Mo_C_ positions of the parent β-octa­molybdate structure. The regioselectivity was achieved by controlled stepwise synthesis *via* vanadium peroxido complexes as precursors. The peroxide-mediated synthesis route (Schwendt *et al.*, 2016 ▸) has already been successfully utilized for the synthesis of several polyoxometalates, such as [H_*x*_V_10_O_28_]^(6–*x*)–^ (Jahr *et al.*, 1963 ▸; Nakamura & Ozeki, 2001 ▸), [H_*x*_PV_14_O_42_]^(9–*x*)–^, Keggin structures [H_3+*x*_PMo_12–*x*_V_*x*_O_40_], and Wells–Dawson structures [H_6+*x*_P_2_Mo_18–*x*_V_*x*_O_62_] (Odyakov *et al.*, 2015 ▸) and [V_12_O_30_F_4_(H_2_O)_2_]^4−^ (Krivosudský *et al.*, 2014 ▸).

## Experimental   

All chemicals were purchased from Sigma–Aldrich (Austria) and used as received.

### Synthesis and crystallization   

For the preparation of K_5_[VMo_7_O_26_]·6H_2_O (**VMo_7_**), K_2_MoO_4_ (1.67 g, 7 mmol) and VOSO_4_·*n*H_2_O (0.2 g, 1.22 mmol) were dissolved in distilled water (40 ml) by heating. HCl (0.7 ml of a 37% *w*/*w* solution) was added. When the temperature reached 80 °C, H_2_O_2_ (0.1 ml of a 30% *w*/*w* solution, 1 mmol) was added and the colour of the solution changed immediately from dark violet to orange. The solution was boiled for 1 min and the pH of the still hot solution was adjusted to 3.1 with 50% KOH solution. The clear-yellow solution was left to crystallize at 18 °C. Yellow block-shaped crystals were filtered off after 2 d, washed with water and ethanol and air-dried (yield 0.47 g, 33%, based on Mo). Elemental analysis (%) for K_5_Mo_7_VO_32_H_12_ (calculated): K 14.0 (13.6), Mo 46.6 (46.6), V 3.43 (3.53).

### Elemental analysis   

Elemental analyses were performed in aqueous solutions containing 2% HNO_3_ using inductively coupled plasma mass spectrometry (PerkinElmer Elan 6000 ICP MS) for Mo and V, and atomic absorption spectroscopy (PerkinElmer 1100 Flame AAS) for K. Standards were prepared from single-element standard solutions of concentration 1000 mg l^−1^ (Merck, Ultra Scientific and Analytika Prague).

### IR spectroscopy   

**VMo_7_** was identified by IR measurement on a Bruker Vertex70 IR Spectrometer equipped with a single reflection diamond-ATR (attenuated total reflectance) unit in the range 4000–100 cm^−1^.

### ^51^V NMR spectroscopy   

^51^V nuclear magnetic resonance spectroscopy measurements of aqueous solutions (with 10% of D_2_O used for locking, at 20 °C) were taken on a Bruker AV II+ 500 MHz instrument operating at 131.60 MHz for the ^51^V nucleus (2000 scans, accumulation time 0.05 s, relaxation delay 0.01 s). Chemical shift values are given with reference to VOCl_3_ (0 ppm) as the standard.

### Refinement   

Crystal data, data collection, structure refinement and software details are summarized in Table 1 ▸. No H atoms were inserted on the free water O atoms due to the disorder and instability of the model. In the case of the disordered groups, one bond was added to the connectivity array (O15*S*—K3). The disordered Mo4*A*/V4 atoms occupying the same position of the POM anion were treated with half occupancies. The O atoms of the solvent molecules (O16*S*, O17*S*, O18*S* and O19*S*) and the partially occupied K3 and K4 atoms and their corresponding *U*_*ij*_ components are of low quality and were forced by the restrained ISOR to affect the standard deviation and approximate the *U*_*ij*_ components to isotropic behaviour.

## Results and discussion   

Upon reaction of the initial H_*x*_VO_4_^(3–*x*)–^ and H_*x*_MoO_4_^(2–*x*)–^ precursors and adjustment of the pH to a certain value, complicated reaction mixtures with several equilibrated species are formed (Howarth *et al.*, 1991 ▸). It was therefore necessary to choose a different synthesis approach that would favour the formation of [VMo_7_O_26_]^5−^. We employed a V^IV^ precursor (VOSO_4_) that forms with molybdate mixed-valence polynuclear deep-blue vanadomolybdates at pH ≃1.5 (Müller *et al.*, 2005 ▸; Botar *et al.*, 2005 ▸). Subsequent addition of hydrogen peroxide resulted in an orange solution formed by immediate oxidation with vanadium peroxido complexes (Schwendt *et al.*, 2016 ▸). The crucial point of the synthesis was the adjustment of the pH of the hot solution to 3.1. This value represents a region where the β-octa­molybdate anion [Mo_8_O_26_]^4−^ is the main species present in the simple molybdate solutions at *c*_Mo_ = 0.1 mol dm^−3^ (Ozeki *et al.*, 1988 ▸). We also obtained different products from solutions with the pH range 1.5–7.0; however, IR and ICP–MS analyses indicated that the products are mixtures and **VMo_7_** can only be obtained as a pure product in the pH range 2.8–3.5. Adjustment of the pH of the cooled solution leads to the formation of precipitates and an obvious reduction of vanadium (formation of a green solution).

**Figure 2:**
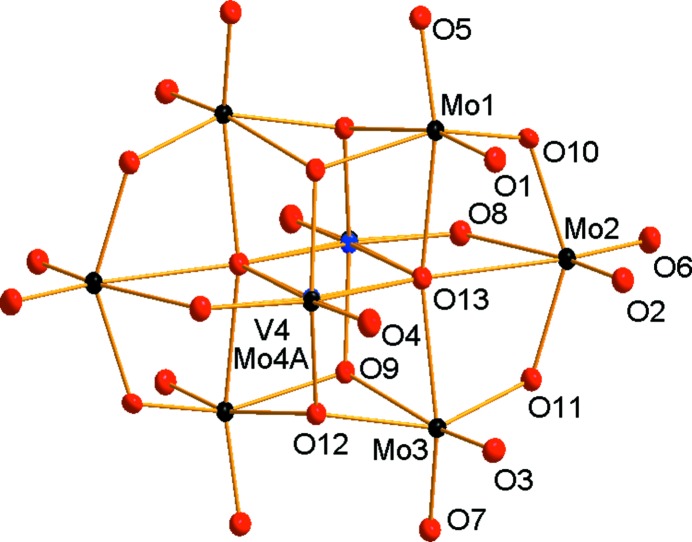
The mol­ecular structure of [VMo_7_O_26_]^5−^ in **VMo_7_**, showing the atom-labelling scheme. Displacement ellipsoids are shown at the 50% probability level. Colour code: Mo black, V blue and O red.

**Figure 3:**
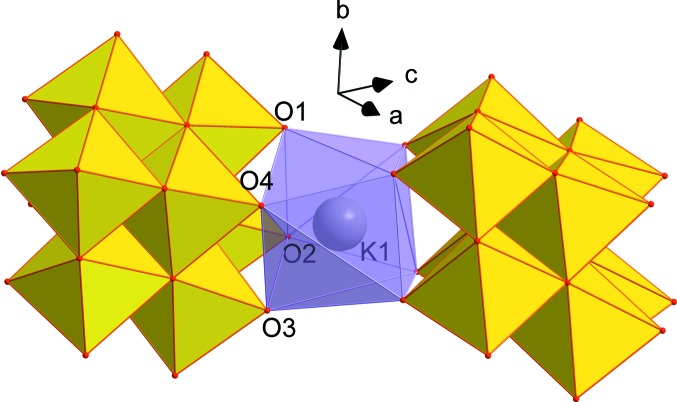
The coordination of the potassium cation K1 by two [VMo_7_O_26_]^5−^ ligands. Colour code: {Mo/VO_6_} yellow octa­hedra and {KO_8_} violet distorted square anti­prism.

**Figure 4:**
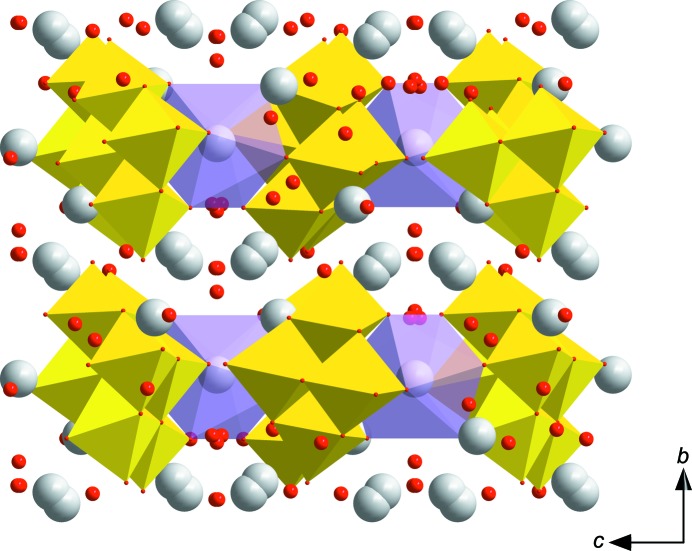
The crystal packing in **VMo_7_**, viewed along the *a* axis. Colour code: {Mo/VO_6_} yellow octa­hedra and {KO_8_} violet distorted square anti­prism. H atoms of water mol­ecules have been omitted for clarity.

The asymmetric unit of **VMo_7_** contains one half of the [VMo_7_O_26_]^5−^ POM anion lying on a centre of symmetry (Fig. 2 ▸). The K^+^ cations which compensate the charge of the anion occupy four positions, one of them at full occupancy (K2) and one disordered over two positions (K3 and K4). The K1 atom is coordinated by two [VMo_7_O_26_]^5−^ anions in an inter­esting fashion, forming an irregular twisted anti­prismatic coordination polyhedron, with K—O distances in the range 2.703 (3)–2.767 (3) Å (Fig. 3 ▸) and without the participation of water mol­ecules. The two square bases formed by oxido ligands O1, O2, O3 and O4 are twisted by approximately 10–13°. The [VMo_7_O_26_]^5−^ anion adopts the expected β-con­formation (compare Figs. 1 ▸ and 2 ▸). The centrosymmetric anion was firstly refined as a pure [Mo_8_O_26_]^4−^ cluster for localization of the V atoms. While the Mo_A_ and Mo_B_ positions showed initial occupancies very close to 1 (≃0.96), significantly lower occupancies of the Mo atoms at the Mo_C_ positions indicated the presence of V atoms which are equally distributed in both symmetrically equivalent positions. The positioning of the V atoms at 0.5 occupancies in the Mo_C_ positions led to a significant improvement of the model. Moreover, it can be seen from Fig. 1 ▸ that the Mo_C_ atoms differ significantly from the Mo_A_ and Mo_B_ atoms in the replacement of one Mo=O bond by a bridging Mo—O bond. Therefore, we consider it reasonable that the V atom occupies preferably position Mo_C_ which is not only crystallographically, but also chemically, markedly non-equivalent to the other positions in the octa­metalate. In this context, we should note for the structure of H_4_K_2_Na_2_(H_2_O)_4_(C_12_H_12_N_4_O_2_)[VMo_7_O_26_]·10H_2_O (Zhao *et al.*, 2018 ▸) that the displacement ellipsoids at the Mo_C_ positions are approximately twice as large as the ellipsoids of the Mo_A_ and Mo_B_ atoms, indicating that the V atom occupies preferably this position also in this structure, although the model was constrained with a statistical distribution of the V atoms throughout the anion. However, the selectivity of vanadium substitution is not known and the existence of such a species might be theoretically possible despite the fact that it was not predicted by speciation (Howarth *et al.*, 1991 ▸). [VMo_7_O_26_]^5−^ consists of eight {Mo/VO_6_} face- and edge-sharing octa­hedra. Except for atoms V4/Mo4*A*, all other Mo atoms are coordinated by two terminal oxido ligands, with shorter Mo=O double bonds in the range 1.601 (5)–1.726 (3) Å, and bridging oxide ligands exhibiting longer bond distances of up to 2.430 (2) Å for the Mo2—O13 bond incorporating the penta­coordinated O atom (see the supporting information for further details). All water mol­ecules exhibit a certain degree of disorder and therefore we do not discuss the hydrogen-bond network. The water mol­ecules complete an irregular coordination polyhedra around the potassium cations, forming a rich polymeric network based on electrostatic inter­actions (Fig. 4 ▸).

**Figure 5:**
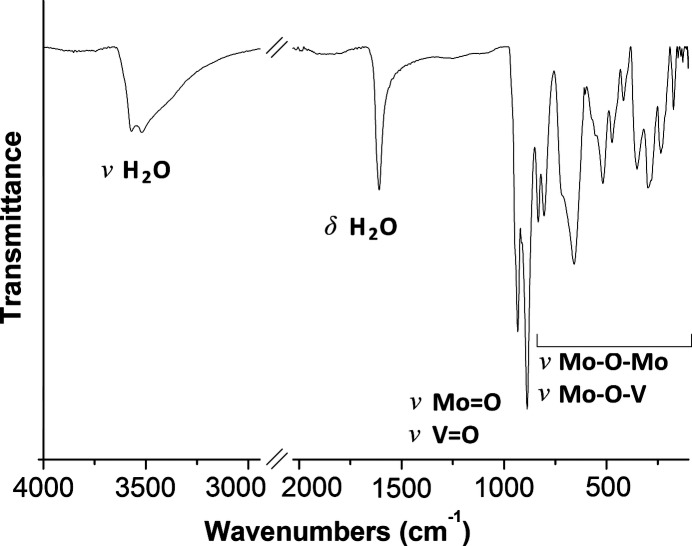
The IR spectrum of **VMo_7_** in the region 4000–100 cm^−1^.

The IR spectrum of **VMo_7_** (Fig. 5 ▸) exhibits bands typical for a β-octa­molybdate structure and the respective bands corresponding to Mo—O or V—O vibrations are not distinguishable. The stretching vibrations of the terminal Mo/V=O units appear at 934 and 888 cm^−1^, whereas the peaks in the region from 470 to 840 cm^−1^ correspond to the anti­symmetric and symmetric deformation vibrations of the Mo—O—Mo and Mo—O—V bridging fragments. The crystallization water mol­ecules exhibit typical bands for valence O—H vibrations and deformation H—O—H vibrations at 1610 and 3540 cm^−1^, respectively.

**Figure 6:**
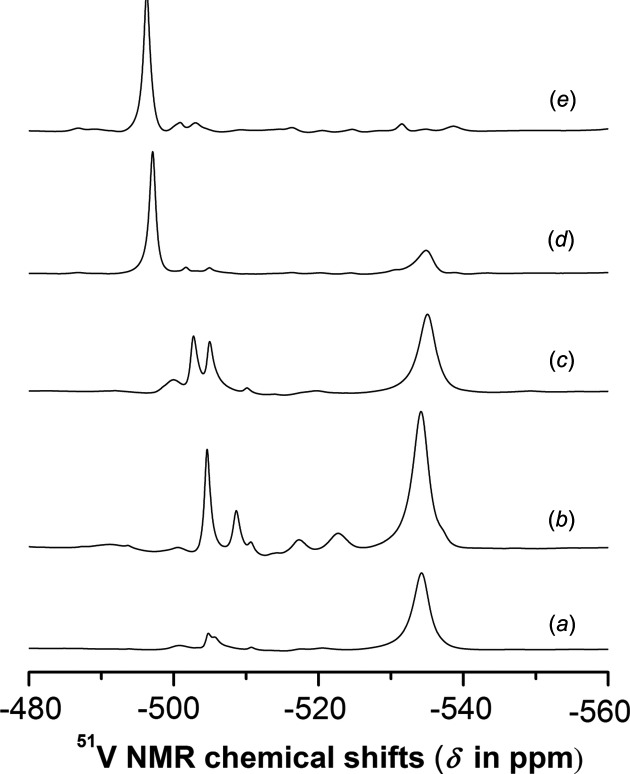
(*a*)/(*b*) The ^51^V NMR spectra of the crystallization solution of **VMo_7_** and (*c*)/(*d*)/(*e*) solutions obtained upon dissolution of crystallized **VMo_7_** at different pH values. Conditions: (*a*) 175 m*M* Mo^VI^, 25 m*M* V^V^, pH = 3.1, 24 h after the synthesis; (*b*) same conditions as (*a*) after another 24 h; (*c*)/(*d*)/(*e*) 10 m*M* V^V^ solution prepared from **VMo_7_** by dissolving it in 0.5 m*M* NaCl at pH values of (*c*) 4.0, (*d*) 5.2 and (*e*) 6.0. The pH was adjusted by the addition of a dilute KOH solution.

We employed ^51^V NMR spectroscopy to inspect the syn­thesis and hydrolytic stability of **VMo_7_** (Fig. 6 ▸). All chemical shifts of the major species were assigned according to a very thorough speciation study based on NMR spectroscopy (^51^V, ^95^Mo and ^17^O) and potentiometric data (Howarth *et al.*, 1991 ▸). In the crystallization solution one day after the synthesis (Fig. 4 ▸ *a*), the [VMo_7_O_26_]^5−^ anion (−534.2 ppm, 95% of V^V^) is dominant, accompanied by a monovanadium-substituted hexa­molybdate [VMo_5_O_19_]^3−^ (−505.0 ppm) and some minor species. After two days (Fig. 4 ▸ *b*), when about 33% of the product has crystallized out, roughly 73% of V^V^ is still consumed in the [VMo_7_O_26_]^5−^ species. Thus, the NMR investigation confirms that a synthetic protocol starting from reduced mixed Mo/V polyoxometalates oxidized by H_2_O_2_ leads almost exclusively to the desired [VMo_7_O_26_]^5−^ anion. The speciation work by Howarth *et al.* proposed that [VMo_7_O_26_]^5−^ should be most stable around pH = 4.2. At this pH value (Fig. 4 ▸ *c*), we observed substantial decomposition of **VMo_7_** into [VMo_5_O_19_]^3−^ (−505.1 ppm) and α-[VMo_7_O_26_]^4−^, a structure that resembles the Anderson–Evans archetype polyoxometalate capped by one Mo=O and one V=O unit (−502.9 ppm). Increased pH (Figs. 4 ▸ *d* and 4 ▸ *e*) results in a profound decomposition of [VMo_7_O_26_]^5−^ into hexa­metalate [V_2_Mo_4_O_19_]^4−^ (−497.0 and −496.3 ppm). The results of the ^51^V NMR measurements showed that once the [VMo_7_O_26_]^5−^ anion is selectively formed from suitable precursors, it stays relatively intact in the solution for at least 24 h. On the other hand, by dissolution of **VMo_7_** in aqueous solution, hydrolysis takes place and the newly formed species, mostly [VMo_5_O_19_]^3−^, α-[VMo_7_O_26_]^4−^ and [V_2_Mo_4_O_19_]^4−^, cannot give rise to the formation of [VMo_7_O_26_]^5−^ even under conditions when the equilibrium of the POM species should be favoured (0.5 *M* NaCl, pH = 4.2).

## Supplementary Material

Crystal structure: contains datablock(s) global. DOI: 10.1107/S205322961900620X/ky3170sup1.cif

Structure factors: contains datablock(s) I. DOI: 10.1107/S205322961900620X/ky3170Isup2.hkl

CCDC reference: 1898165

## Figures and Tables

**Table 1:** Experimental details

Crystal data	
Chemical formula	K_5_[VMo_7_O_26_]·6H_2_O
*M*_r_	1442.12
Crystal system, space group	Monoclinic, *C*2/*c*
Temperature (K)	143
*a*, *b*, *c* (Å)	12.9356 (10), 16.1978 (10), 13.8618 (9)
β (°)	90.962 (4)
*V* (Å^3^)	2904.0 (3)
*Z*	4
Radiation type	Mo *K*α
μ (mm^−1^)	4.06
Crystal size (mm)	0.1 × 0.1 × 0.1

Data collection	
Diffractometer	Bruker D8 Venture
Absorption correction	Multi-scan (*SADABS*; Bruker, 2016 ▸)
*T*_min_, *T*_max_	0.512, 0.564
No. of measured, independent and observed [*I* > 2σ(*I*)] reflections	80344, 4259, 4028
*R*_int_	0.057
(sin θ/λ)_max_ (Å^−1^)	0.705

Refinement	
*R*[*F* ^2^ > 2σ(*F* ^2^)], *wR*(*F* ^2^), *S*	0.027, 0.059, 1.26
No. of reflections	4259
No. of parameters	235
No. of restraints	37
H-atom treatment	H-atom parameters not defined
Δρ_max_, Δρ_min_ (e Å^−3^)	0.64, −0.74

Computer programs: *APEX3* (Bruker, 2018 ▸), *SAINT* (Bruker, 2016 ▸), *SHELXS97* (Sheldrick, 2008 ▸), *SHELXL2018* (Sheldrick, 2015 ▸), *OLEX2* (Dolomanov *et al.*, 2009 ▸), *DIAMOND* (Brandenburg, 2006 ▸), *PLATON* (Spek, 2009 ▸) and *ShelXle* (Hübschle, *et al.*, 2011 ▸).
